# Neural responses to emotional displays by politicians: differential mu and alpha suppression patterns in response to in-party and out-party leaders

**DOI:** 10.1038/s41598-025-92898-6

**Published:** 2025-03-11

**Authors:** Maaike D. Homan, Mohammad Hamdan, Karlijn Hendriks, Diamantis Petropoulos Petalas

**Affiliations:** 1 Department of Political Science, University of Amsterdam, Amsterdam, The Netherlands; 2 Faculty of Social and Behavioural Sciences, Utrecht University, Utrecht, The Netherlands; 3 Brain Research and Innovation Centre MGGZ, University Medical Centre Utrecht, Utrecht, The Netherlands; 4 Department of Psychology, The American College of Greece, Athens, Greece

**Keywords:** Human behaviour, Emotion

## Abstract

The high levels of polarization raise concerns about individuals’ decreased ability to empathize and understand the representatives of political out-groups. As such, our political biases may lead us to misunderstand out-group politicians. In the current study, we examine the mu rhythm, a neural oscillation in the sensorimotor cortex related to the processing and understanding of other people’s actions, intentions and emotions. The mu rhythm is particularly responsive towards the emotional expressions of others and sensitive to social biases. Hence, we examine (1) whether the emotions displayed by politicians lead to more mu event-related-desynchronization (mu-ERD), (2) whether it matters which emotion (angry, happy, neutral) is displayed, and (3) whether neural responses differ when emotions are displayed by politicians we support (in-party politician) compared to politicians we do not support (out-party politician). To test this, we recorded electroencephalogram (EEG) responses during a preregistered Go/No Go mimicry experiment (*N* = 47, Obs = 1104), in which participants are presented with dynamic morphed emotional displays of Dutch politicians (in- and out-party) and non-politicians. We find that politicians emotional displays increase participants’ mu-ERD compared to static neutral displays. Most mu-ERD is found for out-party politicians, especially when angry. In addition, we explored alpha oscillations (related to visual attention), where we find the strongest alpha-ERD for the out-party happy condition. Overall our results suggest that our brain is specifically attuned to process the emotions of out-party politicians.

## Introduction

There are increasing concerns about high levels of polarization in advanced democracies throughout the world^1–3^. A suggested consequence of polarization is that we fail to pay attention, empathize, and understand representatives of the political out-group (i.e., the out-party,^4,5^). This suggested failure to understand politicians from the out-group impedes the development of constructive political dialogue crucial to sustaining a healthy democracy. In this context, effective communication of emotions becomes paramount, particularly for voters seeking to navigate and interpret the emotional cues conveyed by politicians. While research shows that social biases unconsciously affect how we process (emotional) information^6–8^, most polarization research uses self-reported attitudes and feelings about the in-party and out-party^9,10^. As a result, there is a need for more research to examine polarization at the unconscious level. This project aims to investigate how political biases shape the neural processes involved in the processing of emotional cues, particularly when individuals are exposed to emotions expressed by in-party and out-party politicians.

In today’s digital landscape, individuals are frequently exposed to politicians’ emotional appeals through visual communication on social media platforms^11–13^. These emotional appeals act as cues that citizens use to inform their attitudes and voting behavior^14–17^. However, the way individuals understand and respond to the emotions displayed by politicians is affected by our political biases. Research shows that individuals can “catch” the emotions from in-party politicians, whereas the emotions displayed by out-party politicians can lead to opposite emotional reactions^18^. Since this process happens partly unconscious, we extend this line of research by examining whether the emotional displays of in-party politicians are already differently processed at the neural level, compared to the emotions displayed by out-party politicians. In order to test this, we examine a neural signal in the sensorimotor cortex that is related to the processing and understanding of other people’s actions, intentions, and emotions, called the *mu rhythm*^19,20^. The mu rhythm is linked to the facilitation of empathy, prosocial behavior and interpersonal contact^21–23^.

Previous research suggests that the mu rhythm is influenced by group identity, showing increased activation in response to individuals who we see as more similar to us, i.e., our in-group members^21,24,25^. In contrast, some other studies suggest that the mu rhythm might be attuned to the actions and emotions of others who we see as less self-relevant or dissimilar to us, i.e., out-group members^26^. We tested this discrepancy in the literature, by conducting a preregistered (see pre-analysis plan on Open Science Framework at https://osf.io/dsfgv/?view_only=57f9c76b170f4e7f8fac0be8eebf89b2) experiment measuring mu rhythm activation in response to the emotional displays of in- party and out-party politicians. More specifically, we recorded electroencephalogram (EEG) responses during an innovative Go/No Go mimicry task. In addition, we measure the alpha signal at the occipital cortex, which is associated with visual attention^27,28^. Together these mu and alpha measures will increase our understanding of how our political biases influence the neural mechanisms involved when confronted with the persuasive emotional displays of in- and out-party politicians.

### The emotional displays of in- and out-party leaders

During debates, speeches, or other media performances, politicians not only use words to persuade voters, but also signal non-verbal cues, such as their emotional expressions^29–31^. Although individuals should base their evaluations of politicians on rational decision-making, research shows that emotional expressions can influence how we evaluate and support these politicians. For example, politicians using positive emotions are more likely to receive more positive evaluations, compared to politicians who show no emotion^15,32^. Also politicians’ displays of anger can elicit increased positive evaluations^17,33^. When it comes to vote choice, Homan and Schumacher^14^ find that politicians’ emotional expressions can influence the decision to vote for a politician beyond agreeing or disagreeing with the politicians’ position on a certain issue.

Although it is clear that politicians’ emotions are influential, several studies suggest that the persuasive effect of politicians’ emotions depends on *who* expresses this emotion. More specifically, the emotional displays of politicians that we support (our in- party) could be received differently than the emotions expressed by politicians we would never vote for (the out-party;^18,33,34^). For example, people unconsciously mimic the emotions of politicians, but only if this is a happy expression from an in-party politician^18,35^. In contrast, Stapleton and Dawkins^33^ find that especially the anger displayed by in-party politicians elicits emotional contagion in supporters and leads to increased willingness to vote. Moreover, to what extent our politically polarized beliefs influence our response towards the emotional displays of politicians remains largely unclear, especially because part of this process happens unconsciously. A growing body of neuroscience work suggests that such biases in social cognition may already manifest at the level of neural activation, before they show at the level of behavior and conscious awareness^21,36^. Also in context of politics, different neural activation is found in response to viewing in-party versus out-party politicians’ faces^37^.

In the current study, we specifically examine how our brain processes the emotional expressions of in-party versus out-party politicians. Using electroencephalogram (EEG), we can measure neural oscillations related to the processing, attention, and understanding of the emotions of others^20,38^, which helps us understand how deep polarization runs and specifically how political biases can alter the way we respond and understand the emotional displays of politicians.

### The mu rhythm

More specifically, we analyze mu rhythm, a series of neural oscillations in the sensorimotor cortex with a frequency of 8–13 Hz^39^. The mu rhythm is typically measured over the central electrodes using electroencephalography (EEG)^20,38^. During rest, a population of neurons in the sensorimotor cortex fire synchronously, leading to so-called high mu power. During both the execution and the mere perception of an (motor) action (e.g., hand movements, grasping an object, emotional expression), sensorimotor cortical activity increases, leading the neurons to fire out of synchrony, resulting in decreased mu power—or mu desynchronization (also called *event-related desynchronization*—ERD,^20,39^).

In general, scholars suggest that the mu rhythm contributes to understanding the actions, intentions, and emotions of others. For example, mu rhythm desynchronization is related to perspective taking^40,41^, pain empathy^24^, intention understanding^42^ and emotion recognition^41,43,44^. The mu rhythm is therefore seen as an important neural mechanism for facilitating empathy, prosocial behavior and interpersonal contact^21,22^. Not surprisingly, this mu-ERD in response to emotional expressions is already present at an early age of development (30 months;^45^). Moreover, the mu rhythm is particularly responsive to facial expressions. For example, Moore et al.^43^ find that the mu rhythm is specifically activated in response to photos of people expressing certain emotions, but not in response to, for example, pictures of buildings or visual noise. In addition, the mu rhythm is more responsive to emotional expressions than to emotion words^44^. Some research suggests that especially ambiguous emotional expressions such as a neutral expression or a happy expression with a tear elicit strong mu-ERD compared to more clear cut expressions (e.g., smiling face)^45–47^.

Besides it’s sensitivity towards emotions, mu-ERD is modulated by group identity. Gutsell and Inzlicht^21^ were one of the first to show that the mu rhythm is more responsive when watching racial in-group members perform an action, compared to racial out-group members. This in-group bias is stronger for individuals with high racial prejudice. Other studies further show that the mu rhythm is more strongly activated towards others who are similar to us^24^ or are perceived as more human^23^. In addition, brain imaging studies show that brain areas related to empathy and mental simulation exhibit more activation in response to in-group members in pain^25^ or expressing emotions^48^, compared to out-group members. Overall, this line of research suggests that the mu rhythm is especially attuned to the people we are more likely to interact or empathize with.

Following this line of reasoning, we examine whether political preferences affect mu suppression. Namely, individuals can feel strongly about the political parties or leaders they support (the in-party) and would not support (out-party). Identification with a political party can result in feelings of belongings, attachment and commitment to the political in-party^49^. In contrast, people can also experience strong negative emotions regarding certain politicians or one’s out-party^50,51^. Taken together, since some of the mu rhythm research suggests that mu-ERD is especially responsive towards our in-group^21,48^, we expect the emotional displays of in-party politicians to elicit more mu-ERD compared to out-party politicians (**H1**).

Although most literature supports an in-group bias in mu rhythm activity, there are some cases in which the out-group elicits more mu rhythm activity compared to the in-group. For example, Losin et al.^26^ find more mu-ERD to racial out-groups during an imitation task. The authors suggest that the enhanced activity reflects the increased neural ‘costs’ (p. 3602^26^) associated with trying to imitate an out-group member. Because out-group members are perceived as less self-relevant and more dissimilar, their actions and emotions tend to be more unpredictable, leading to increased neural effort^26^. This pattern is also reflected in research on facial processing. For example, Lee et al.^52^ found that out-group faces require more intensive neural processing, while in-group emotional faces are processed more automatically and with less cognitive effort. Moreover, unfamiliarity with the intentions and emotional expressions of out-group members necessitates greater cognitive resources to interpret their behavior^53,54^. Following this line of reasoning, the increased neural effort required to understand out-group members may lead to greater mu-ERD when processing the emotions of out-party politicians compared to in-party politicians (**H2**).

When it comes to the specific emotion categories, several expectations can be described. First, happy expressions typically signal affiliative intent and stimulate approach behaviors^55^. Moreover, happy expressions are more likely to be mimicked compared to more antagonistic emotions such as anger^35,56^. In other words, happy expressions are easier to empathize with compared to negative emotions. If we follow the literature suggesting an in-group advantage in mu rhythm activation (H1), we can expect that the happy expressions of in-party politicians elicit mu-ERD, compared to angry or neutral expressions (**H3**). However, following the research showing more mu-ERD for the out-group (H2), we expect the happy expressions of out-party politicians to elicit more mu-ERD compared to the other emotion conditions (**H4**). Namely, due to our negative association with an out-group, people tend to associate angry emotions to out-group members. Positive emotions displayed by an out-group member are therefore more unexpected^57,58^, possibly requiring stronger mu-ERD to resolve this ambiguity.

Traditionally, mu-ERD has been associated with a form of mental simulation, whereby our brain simulates an action we observe in others in the same brain areas as when performing the action ourselves. This brain network has been labeled as the ‘mirror neuron system’ (MNS;^59^). Through activation in the MNS, observers unconsciously mirror the actions and emotions observed in others, leading both individuals to be in synchrony with each other^60,61^. However, this interpretation of automatic mirroring by the MNS has become a contested issue in the literature (see^20,38,62^). Recent research suggest that instead of automatic mirroring, the network is activated to understand the actions and emotions of others in order to plan an adequate response, which is not necessarily a mirroring response^19^.

At the time of preregistration, we based one of our hypothesis on the (now contested) literature of the mirror neuron system (see reviews by^19,63^), assuming that observing emotional expressions automatically elicits mu-ERD, so also when displayed by politicians (**H5**, both in- and out-party politicians). Moreover, as described above, the mu rhythm does not necessarily lead to mirroring and seems to be particularly responsive to people who we would like to affiliate with, such as in-group members^21,24^. However, when it comes to politics, people can experience high levels of distrust towards the political elites, assuming that the politicians mostly act based on self-interest^64,65^. In addition, Homan et al.^18^ find that people do not automatically mimic the emotions of politicians. They only do so under specific circumstances. Hence, we preregistered the competing hypothesis that the emotional displays of politicians (both in- and out-party politicians) do not elicit mu-ERD (**H6**; for better readability of the paper we switched the numbering of our hypotheses that we preregistered and instead of ‘MNS activity’ use the term ‘mu-ERD’).

Finally, besides our preregistered expectations regarding mu rhythm activity, we also explore the alpha rhythm in the occipital areas. Namely, the oscillatory activity of the mu rhythm that falls within the alpha-band range (8–13 Hz). Hence, several scholars have pointed out that it is important to distinguish mu and alpha signals, since the two can sometimes be conflated^20,66^. The alpha signal measured over the occipital regions is associated with visual attention^27,28^, which facilitates information processing^67^. Similar to the mu rhythm, alpha power is high when in rest, due to synchronous firing of neurons. When activated, neurons in the occipital region fire out of synchrony, leading to decreased alpha power, or alpha desynchronization (alpha-ERD). In the current study, we explore the role of alpha-ERD in response to the emotional displays of politicians. This allows us to disentangle mu from alpha rhythm in processing facial emotions, and to provide important insights into the underlying mechanism of attention when observing the emotional displays of politicians.

### The present study

In sum, the aim of the present study is to examine (1) whether the mu rhythm is activated in response to the emotional displays of politicians, and (2) whether this mu rhythm activity differs for in-party (i.e., the party or politician you are most likely to vote for) versus out-party politicians (i.e., party or politician people are least likely to vote for) emotional displays. In addition, we explore alpha-ERD as a measure of visual attention, in response to politicians’ emotional displays.

To test this, we use a Go/No Go mimicry task, similar to Krivan et al.^47^. The common standard within the mu rhythm literature is to measure mu-ERD while participants observe and execute (i.e., mimic) a certain movement^47,68^. The rationale is that both the passive viewing of an action and the execution of that action elicit similar brain responses^59^. Following this research tradition, we instructed participants to mimic the emotional expressions of in-party politicians, out-party politicians, and unknown individuals (non-politicians), while recording mu rhythm activity using EEG measurement. To limit the demands of the experiment for the participants, we included only an execution task and no passive observation task. In addition, passive observing of facial expressions can elicit mimicking responses in participants^69^. These facial movements could create artifacts in the neural signal. Hence, we only included a Go/No-Go mimicking task following Krivan and colleagues^47^.

**Fig. 1:**
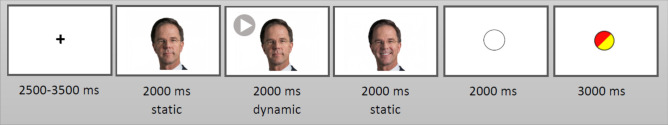
An example of a trial showing a politician (Mark Rutte of the VVD, Credit: Ministerie van Buitenlandse Zaken, licensed with CC BY-SA 2.0). The 6000 ms video consists of a static 2000 ms neutral expression, a dynamic 2000 ms happy, and a static 2000 ms happy expression. The video is followed by the presentation of a circle for 2000 ms, which then turns either yellow (GO) or red (NO-GO) for 3000 ms. Participants are instructed to keep their faces still during the trials. Only when the circle turns yellow they should move their faces to mimic the emotional expression observed in the first part of the trial.

More specifically, in this Go/No-Go mimicking task, we present participants with dynamic morphed emotional expressions (i.e., a neutral mouth opening movements, angry- and happy facial expressions, for more information see the Method section) of in-party and out-party politicians and unknown individuals (non-politicians). Participants are asked to first observe a dynamic emotional display and then either mimic (Go) or refrain from mimicking (i.e., keep their face still; No-Go) the display (^47^, see an example trial in Fig. 1). Participants are specifically instructed not to move their face (i.e., keep it relaxed) and only move their face when the ‘mimicking’ cue appears (Go). Participants practice this in several practice rounds with guidance from a research assistant. The EEG measurement obtained during the observation of dynamic emotional display, but not during the mimicry task, is used for analysis. This way, the neural response contains the intention to mimic, without the interference of motor movement during the mimicking task (which could create artifacts). By using this Go/No-Go procedure, mimicry during the observation of emotional displays is minimized^47^ and allows us to gain more insights into how political biases affect how individuals process the emotional displays of politicians at a deep and early level of processing.

## Results

**Fig. 2:**
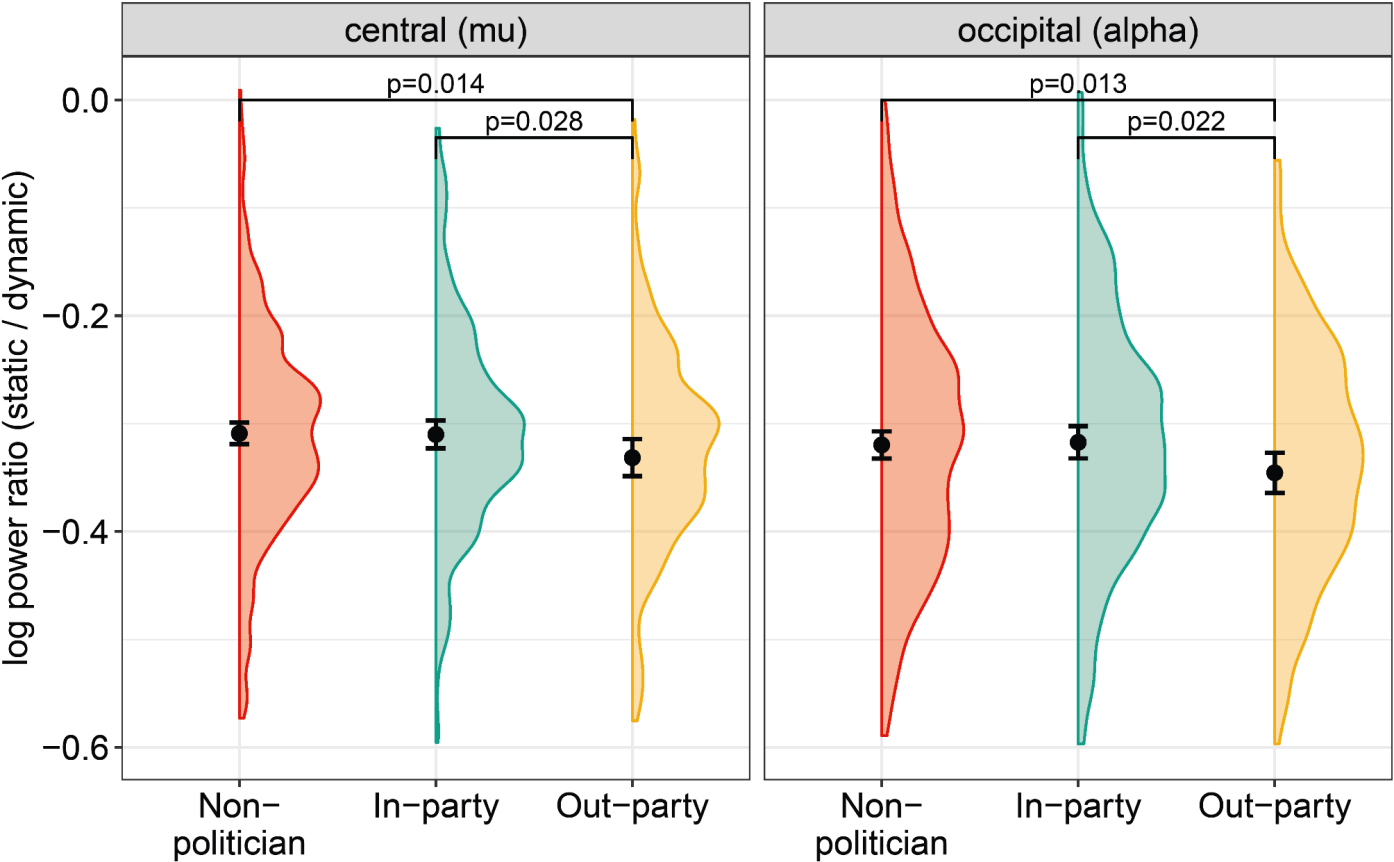
Log power (spectral density) ratio (static phase first 2000 ms/dynamic phase second 2000 ms) of 47 participants (Obs = 1104) over the central electrodes (C3, C4, mu rhythm) and occipital electrodes (O1, O2, alpha signal) for the different politician conditions: non-politician (in red), in-party politician (in green), and out-party politician (in yellow). Black dots represents the mean estimate per group, the black whiskers the 95% confidence intervals, and the clouds the sample distributions. P-values are given for significant differences between conditions.

To test our hypotheses, we use both OLS regressions and Bayesian paired sample t-tests (see pre-analysis plan for details). First, we examine whether in-party politicians elicit different levels of mu event-related desynchronization (ERD) compared to out-party politicians over the central electrodes. More specifically, we test hypothesis 1 (in-party > out-party) and hypothesis 2 (in-party < out-party) by conducting OLS regressions with standard errors clustered at the individual level and with mu-ERD as dependent variable (see control variables in pre-analysis plan and Supplementary Material). By collapsing together all emotion conditions (angry, happy, neutral), we find that the out-party politicians elicited more mu-ERD compared to in-party politicians (*β* = − 0.021, *SE* = 0.010, *p* = 0.028, *d* = 0.19) and compared to non-politicians (*β* = − 0.022, *SE* = 0.010, *p* = 0.014, *d* = 0.19), which can be seen in the left panel of Fig. 2 (see Tables S1–2 for descriptive statistics, Table S5–6 for regression results, and Table S17–19 for Bayes Factors in Supplementary Material). The Bayes Factors indicate no evidence for the null hypothesis (i.e., that in-party and out-party condition are equal, BF_01_ < 1) and anecdotal evidence for the alternative hypothesis (i.e., that the out-party elicits more mu-ERD than the in-party condition, BF_10_ = 2.016). Based on these results, we find more evidence favoring hypothesis 2, namely that the emotional displays of out-party politicians elicit more mu-ERD compared to the in-party.

**Fig. 3:**
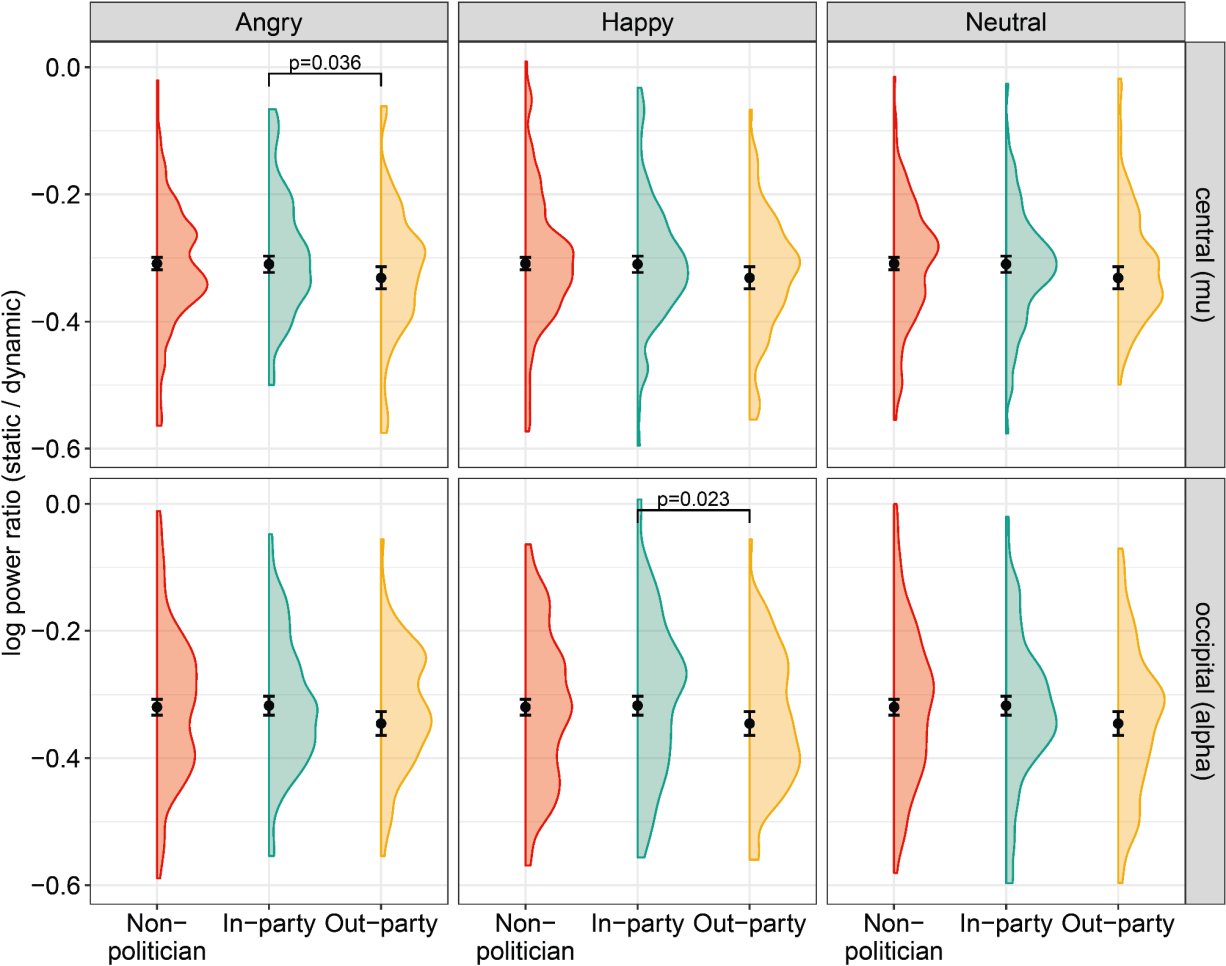
Log power (spectral density) ratio (static phase first 2000 ms/dynamic phase second 2000 ms) of 47 participants (Obs = 1104) over the central (mu) and occipital (alpha) electrodes for each politician condition: non-politician (in red), in-party politician (in green), and out-party politician (in yellow) per emotion condition (left: angry, middle: happy, right: neutral). Black dots represents the mean estimate per group, the black whiskers the 95% confidence intervals, and the clouds the sample distributions. *P* values are given for significant differences between conditions.

Second, we specifically examined differences between emotion conditions within each group condition. First, we expected in-party happy displays to elicit more mu-ERD compared to neutral and angry in-party displays (H3). However, we did not find any differences between the emotion conditions of the in-party politician displays (neutral versus happy: β = 0.002, SE = 0.014, *p* = 0.890; neutral versus angry: β = 0.008, SE = 0.013, *p* = 0.564). Second, we expected out-party happy displays to elicit more mu- ERD than neutral or angry out-party displays (H4). Within the out-party conditions, we again find no differences between emotion conditions (see Table S7 and Figure S3 in Supplementary Material; neutral versus happy: *β* = -0.024, *SE* = 0.020, *p* = 0.236; neutral versus angry: *β* = − 0.024, *SE* = 0.019, *p* = 0.221). The Bayes Factors indicate moderate (BF_01_ > 3) to strong (BF_01_ > 10) evidence for the null hypothesis (see S18 in Supplementary Material), so we reject both H3 and H4. However, when looking at differences between the politician conditions within each emotion category, we find that in the angry condition, the out-party politicians elicit statistically significantly more mu- ERD compared to in-party politicians (*β* = − 0.034, *SE* = 0.016, *p* = 0.036, *d* = 0.32, BF_10_ = 2.58, see left upper panel of Fig. 3 and Table S5 in Supplementary Material). There are no differences in mu-ERD between the groups in the happy and neutral conditions (neutral in-party versus out-party: *β* = − 0.002, *SE* = 0.017, *p* = 0.890, happy in-party versus out-party: *β* = − 0.028, *SE* = 0.018, *p* = 0.116).

Third, we test whether the emotional display of politicians (in-party and out-party politicians together) elicit mu-ERD (H5 & H6). We do this by conducting paired sample t-tests and calculating Bayes Factors between the static phase (neutral picture) versus the dynamic phase (moving emotional or neutral expression) for the politician conditions. Overall, the dynamic phase elicited more mu-ERD (*M* = − 0.256, *SD* = 0.842) compared to the static phase (*M* = 0.082, *SD* = 0.855) in the politician conditions (in-party and out-party politicians conditions together: *M*_*dif*_ = 0.338, *t*(4214) = 47.830, *p* < 0.001, *d* = 2.32). The Bayes Factor indeed indicates no evidence for the null hypothesis (BF_01_ < 0.001) and strong evidence for the alternative hypothesis (BF_10_ > 10). The dynamic phase elicited more mu-ERD in all other conditions as well, see Tables S11–13 for all t-test results and Tables S17–19 for Bayes Factors in the Supplementary Material. Based on these results, we accept hypothesis 5, politicians’ emotional displays elicit mu-ERD.

Finally, we examine the alpha-ERD in response to the emotional displays of politicians. Overall, the correlation between mu and alpha is *r* = 0.48 (*p* < 0.001). When adding alpha activity as control variable in our models, alpha activity is a statistically significant predictor of mu rhythm activity (*β* = 0.402, *SE* = 0.079, *p* < 0.001). In addition, when controlling for alpha activity, the difference in mu-ERD is between out-party and in-party politicians is no longer statistically significant, but remains in the same direction (*β* = − 0.010, *SE* = 0.008, *p* = 0.234). Furthermore, there is no significant difference in neural activity based on location (central versus occipital; *β* = 0.007, *SE* = 0.07, *p* = 0.332; see Table S20 in Supplementary Material). These findings indicate that mu and alpha results are related.

When using alpha activity as dependent variable in our models, we indeed find similar results except one. First, similar to mu, participants also exhibit more alpha-ERD in response to the out-party politicians compared to in-party politicians (*β* = − 0.028, *SE* = 0.012, *p* = 0.022, *d* = 0.21, BF_10_ = 3.80) and compared to non-politicians (*β* = − 0.026, *SE* = 0.010, *p* = 0.013, *d* = 0.20, BF_10_ = 2.68, see right panel of Fig. 2 and Tables S8–10 in Supplementary Material). Second, there are no differences between emotional displays within the in-party and out-party conditions (see lower panels of Figure S3). In contrast with the mu findings, happy out-party politicians elicit more alpha-ERD compared to happy in-party politicians (*β* = − 0.046, *SE* = 0.019, *p* = 0.023, *d* = 0.35, BF_10_ = 3.72, see middle lower panel of Fig. 3). Lastly, similar to mu, the dynamic phase of all politician and emotion conditions elicit more alpha-ERD compared to the static phase (*M*_*dif*_ = 0.353, *t*(4214) = 48.876, *p* < 0.001, *d* = 2.37, see Tables S13–14 in Supplementary Material).

## Discussion

The aim of the current study was to examine whether neural activity differs in response to the emotional displays of politicians we support (in-party politician) versus politicians we are least likely to vote for (out-party politicians). More specifically, we measured mu rhythm activation, a neural oscillation related to the processing and understanding of the actions, intentions and emotions of others^19,24,41,43^. Using a Go/No Go mimicry task, we indeed find that when participants are observing dynamic emotional displays of politicians (with the intention to potentially mimic them in a later stage), the emotional displays of politicians elicit mu event-related desynchronization (mu-ERD). Interestingly, we find that the strongest mu-ERD for the emotional displays of out-party politicians, compared to in-party politicians and non-politicians. Especially when displaying anger, out-party politicians elicit more mu-ERD than in-party politicians. In addition, we explored the alpha activity over the occipital electrodes, which is related to visual attention^27,28^. Similar to mu, we find more alpha-ERD in response to the out-party emotional displays. However, in contrast to mu, most alpha-ERD was found in response to out-party happy displays. Overall this study shows that the neural processing of emotional displays of politicians is affected by our political biases.

What do these findings imply? Several interpretations are possible. First, our results align with previous literature showing increased mu rhythm activation in response to out-group members^26,70^. This line of research suggests that because out-group members are less self-relevant and more dissimilar to the observer, this leads to increased neural processing^26^. However, other scholars find that in-group members elicit more mu-ERD, which is seen as an indication of increased empathy^21,23,24^. One explanation for this discrepancy, could be the difference in experimental designs. Namely, Losin et al.^26^ find that when participants merely observe in- and out-group members, more activation was found for the in-group members. However, when asked to engage in an imitation task, more activation was found for the out-group. In similar fashion, Gutsell et al.^70^ find that when actively engaging participants in a perspective taking task with the out-group, mu-ERD is stronger for the out-group. Since our experimental task also involves active participation by mimicking the emotional displays, this could have resulted in more mu-ERD for the out-party politicians. This might indicate an increased motivation to understand the out-party when asked to actively engage with the emotional displays. Future research should examine whether this increased mu-ERD for out-party politicians also holds when participants are only asked to merely observe the emotional displays.

However, dislike for the out-party is a deeply rooted feeling. An extensive line of research in political psychology shows that individuals experience strong negative (even disgust) feelings towards out-party politicians, both consciously and unconsciously^18,50,51,71^. Hence, using political parties as in- or out-group might work differently compared to previous research studying the effect of group identity on mu rhythm activation with for example ethnicity^21,26^. Moreover, the finding of increased mu rhythm activation in response to angry out-party politicians, aligns with a mimicry study of Homan et al.^18^. Namely Homan et al.^18^ find that people mimic the happy expressions from in-party politicians, but show a strong reactive response towards out-party politicians, especially when they express anger. According to the mimicry literature, mimicking can be seen as an empathetic response with the observed emotional expression, whereas a reactive response is an emotional response in reaction to the observed expression, often occurring when an emotion is expressed by an out-group member^8,72^. Mu rhythm activity has been previously associated with facial mimicry (although the exact nature of this relationship is debated in the literature;^73–76^). Moreover, our finding of stronger mu-ERD towards the out-party angry condition may not necessarily reflect increased empathizing, but could rather be interpreted as increased engagement to understand the intentions of the other (out-party), in order to plan an appropriate (potentially reactive or polarized) response^19^.

This increased engagement with the out-party, is further supported by our exploratory findings of increased alpha activity in the occipital region in response to the out-party politician conditions compared to the in-party politicians and unknown individuals (non-politicians). Namely, besides our preregistered hypotheses, we explored the effect of politicians’ emotional displays on alpha activity at the occipital region, associated with visual attention^27,28^. We find stronger alpha-ERD in response to happy out-party politicians compared to happy in-party politicians. This findings aligns with research that shows heightened attention towards out-group members^77^. In addition, people tend to associate out-group members with negative emotional expressions, such as anger^78^. A happy expression from an out-group member is therefore unexpected and can lead to increased neural processing, as shown by Gamond et al.^58^. Similarly, the increased alpha-ERD in response to the happy out-party condition might thus reflect the allocation of attentional resources towards this incongruency^79^. This also resonates with research in political psychology, showing that especially messages from the out-party or messages incongruent to one’s world view, receive more engagement^80^.

While these findings offer important insights into the neural processing of the emotional displays of politicians, several limitations of the current study should be acknowledged. First, we investigated the activation of the mu rhythm with a mimicking task, common within the mu rhythm literature^47,68^. However, observing facial expressions (before mimicking them), can unconsciously elicit mimicking in the face^69^. Despite efforts to limit artifacts from mimicry movements, such as the Go/No Go nature of the task, the practice rounds, and the removal facial movement signals in the pre-processing procedure, artifacts in the EEG signal due to mimicking movements cannot be ruled out completely. Future research should therefore include facial EMG measures to better control for mimicking and furthermore aim to invent alternative experimental tasks that are more suitable for mu rhythm facial expression research. Second, another limitation of this type of research design, is that it is hard to distinguish mu and alpha activity. We indeed find that mu and alpha activity are partly related in our study. Alpha activity is highly sensitive to changes in attention due to for example changes in visual representations^27,28^. However, due to the use of a static neutral display as baseline, before the dynamic emotional display, the difference in (visual) attentional demands are solely based on the group condition, since the movement is similar across all conditions. Future research should aim to further disentangle the relationship between mu and alpha (a commonly debated issue in the literature see^20^).

Another limitation of our research design is that we did not include a non-biological movement condition in our experiment. Future research could include a non-biological movement condition (see, for example, the scrambled face condition in^45^) to further isolate mu rhythm activity from general visual or motion demands and demonstrate its specificity for human-related or empathy-driven actions. Finally, another interesting avenue for future research could be to include a non-politician leader condition of unknown individuals that have high status positions (e.g., CEO’s). Previous research has found that status can influence imitating processes^81^. Hence, future research could investigate whether our findings extend to in-group/out-group leaders or is specific for politicians.

Overall, our study shows that when asked to actively engage with the emotional displays of out-party politicians, neural activity is increased compared to in-party and non-politicians. In other words, the current study shows that political preferences influence how our brain processes the emotional displays of politicians. More research is needed to explain how this biased neurological processing of the emotions of politicians influences later affective, cognitive, and behavioral responses and potentially fuels polarization in our society.

## Method

### Design

The present study has been approved by the Ethics Review Board of the University of Amsterdam (#2021-AISSR-13386) and was performed in accordance with the psychophysiological research regulations. Data was collected from February to May 2022 (preregistered November 2021).

This experimental lab study consisted of a 3 (emotion: neutral, happy, and angry) by 3 (character: in-party politician, out-party politician, and non-politician) within subject design. Participants were exposed to the emotional displays of 2 in-party politicians and 2 out-party politicians. To validate the mu suppression signal, we included 4 emotional displays of non-politicians (two male and two female subjects). These stimuli are taken from the Amsterdam Dynamic Facial Expression Set (ADFES,^82^) and have been shown to elicit mu suppression before^45^. The non-politician conditions furthermore allow us to compare the effects of the emotional displays of politicians to non-politicians on mu rhythm activity. In total, one experimental block consists of 24 trials (8 different characters with each 3 different emotions), which is repeated 4 times (96 trials in total) with breaks in between each block.

### Sample

We conducted an a-priori power analysis using Power Contour Estimation^83^, based on an experimental design including 96 trials. We calculated the sample size necessary to detect an interaction effect based on the results of a pilot from June 2021, and data from Karakale et al.^46^, who used a similar design. We found that with power set at 80%, we would require a sample size of 48 participants (for more details see pre-analysis plan or Supplementary Materials).

We collected data from N = 52 participants and excluded five of them, one because they requested to stop after starting the experiment and four for being left handed (a-priori grounds for exclusion). We were left with data from N = 47 (age: *M* = 22.81, *SD* = 6.84, 32 females) Dutch right-handed participants, who participated for a monetary reward or course credit. Out of these participants, 39% considered themselves left-leaning, 40% reported themselves to be non-religious and 39% as liberal. See Tables S3–4 in Supplementary Material for participants’ in- and out-party choices.

### Procedure

After signing informed consent, participants were presented with two experiments in random order (of which one is unrelated to the present study). For the current experiment, we used Neurobs’ Presentation software (Version 18.0, Neurobehavioral Systems, Inc., Berkeley, CA) to present the stimuli. The participants were instructed not to move their face and to remain motionless until otherwise instructed.

The trial sequence began with a fixation cross. To avoid anticipation of the stimuli, we randomly varied the fixation cross duration to last between 2500 and 3000 ms ^47^. Next, and following a similar approach as in^46^, a 6000 ms video was shown to the participant. The first 2000 ms of the video presented a static picture of a character exhibiting a neutral expression. This period was used as a baseline in the analysis, as Hobson and Bishop^84^ have demonstrated that an within-trial static baseline shows the most reliable pattern of mu suppression. In the next 2000 ms, the expression changed from a static neutral expression to one of the emotion conditions. Finally, the changed expression remained on screen for another 2000 ms (see Fig. 1).

After the 6000 ms video stimulus, subjects were asked to follow a Go/No-Go mimic procedure, similar to the study of Krivan et al.^47^. After observing the dynamic emotional displays, subjects were presented with an empty black-lined circle for 2000 ms, which would then change into either yellow (Go) or red (No-Go) for another 3000 ms (see Fig. 1). Participants were instructed to either mimic the expression (which they observed before the circle appeared), when the circle turned yellow, or actively inhibit mimicry (i.e. keep their face still) when the circle turned red. Participants practiced the trail sequence for several rounds. Research assistants emphasized to the participants to keep their face still and relaxed until the ‘Go’ cue appeared. Furthermore, the research assistants observed the participants through a camera during the complete experiment, to make sure the participants were following the instructions. The EEG measurement obtained during the presentation of the video, but not during the mimicry task, was used for analysis. This way, the neural response registered the intention to mimic, without the interference of motor movement during the mimicking task (which could create artifacts). All stimuli were randomly presented once at every block of trials and each block was repeated four times.

### Stimuli

The stimuli consist of three different characters: in-party politicians, out-party politicians and non-politicians portraying either happy, angry, or neutral facial expressions. Non-politicians with neutral facial expressions were taken from the Amsterdam Dynamic Facial Expression Set (ADFES,^82^). Portraits from Dutch party leaders were taken from www.tweedekamer.nl or their party website. We included the 17 party leaders in the Dutch parliament at the time of the experiment, in early May 2021. The party leaders at the time of data collection were Mark Rutte (VVD), Geert Wilders (PVV), Wopke Hoekstra (CDA), Sigrid Kaag (D66), Jesse Klaver (GroenLinks), Lilian Marijnissen (SP), Lilianne Ploumen (PvdA), Gert-Jan Segers (ChristenUnie), Esther Ouwehand (PvdD), Liane den Haan (Fractie Den Haan), Kees van der Staaij (SGP), Farid Azarkan (Denk), Thierry Baudet (FvD), Laurens Dassen (Volt), Joost Eerdmans (JA21), Caroline van der Plas (BBB), Sylvana Simons (BIJ1).

Subsequently, both the expressions of the politicians and non-politicians were manipulated to express the emotions of interest in accordance with the study of Homan et al.^18^. In order to create a dynamic change in facial expression—to increase the probability to elicit most MNS activity^85^—the ‘Batch > Multiple Continua’ function in WebMorph^86^ was applied and then saved as video using Microsoft Photos. The happy facial expression always included smiling, teeth-showing faces. For the neutral condition, we followed a similar approach as Rayson et al.^45^, we created an opening and closing of the mouth movement, which has been validated as emotional neutral facial movement^45^.

### In-party and out-party assignment

The experiment was conducted in the Netherlands, a country with a multi-party system with seventeen parties in parliament. At the start of the experiment, we asked participants about their in-party: “which of the following two parties has the highest probability of receiving your vote during the next national elections?” (with a list of all 17 parties as answer options in random order). For the out-party, participants were asked “which two parties will certainly NOT receive your vote during the next national elections?” (with again the 17 parties in random order). The 17 parties in the Dutch parliament at the time of data collection: VVD, PVV, CDA, D66, GroenLinks, SP, PvdA, ChristenUnie, PvdD, 50Plus, SGP, Denk, FvD, Volt, JA21, BBB, BIJ1—for a similar approach, see Bakker et al.^87^.

### EEG data acquisition

EEG was recorded at a sampling rate of 512 Hz without online filters, using 64- channels with a BioSemi ActiveTwo system (DC amplifier, 24-bit resolution). Electrodes were placed according to the international 10–20 EEG system. Additional electrodes Common Mode Sense (CMS) and Driven Right Leg (DRL) served as ground electrode and the reference electrodes were located bilaterally on the mastoid bones. Two additional electrodes were placed, one next to and one above the left eye, to correct for eye movements. Electrolytic gel was applied before the start of data recording. Electrode impedance was kept below 50 kΩ. The lab-assistants monitored the subjects and measurement recordings in the adjacent room via computers and camera during the duration of the experiment.

### EEG pre-processing

EEG processing was performed using EEGLAB, an open-source MATLAB toolbox^88^. The pre-processing procedure consisted of several steps. First, the continuous data was down sampled to 250 Hz and high-pass filtered at 0.1 Hz for each subject. Line noise occurring at the harmonics of 50 Hz was removed by low-pass filtering the data at 40 Hz. Second, bad channels were identified and interpolated by using the EEGLAB pop_rejchan function (absolute threshold or activity probability limit of 5 SD, based on kurtosis). Then, 6000 ms epochs were created according to the stimulus duration, to remove between-session data. The EEGLAB pop_autorej function with default settings was applied for automated artifact epoch detection and rejection. Infomax ICA (Independent Component Analysis) was performed on all pre-processed datasets using EEGLAB default settings. Based on the ICA, we rejected eye and muscle movement artifacts for each participant. Finally, data was re-referenced to the average of all electrodes.

Further pre-processing steps followed the approach of Karakale et al.^46^. Epochs were extracted for each condition, resulting into early epochs of 800–1900 ms (period of 1100 ms) corresponding to the static video; and 1950–4000 ms for the dynamic epochs (period of 2050 ms), corresponding to the dynamic video. Data were extracted for the mu/alpha band (8–13 Hz frequency) at five central electrodes C3, C1, Cz, C2 and C4; and at three occipital electrodes O1, Oz and O2. Subsequently, Fast Fourier Transform (FFT) was used for all eight electrodes to calculate the power spectral density (PSD) in each trial for the static and dynamic epochs, separately.

Next, a ratio value of the dynamic epoch PSD relative to the static epoch PSD was calculated for all electrodes and trials in every participant. The PSD ratio value was used as the measure for mu rhythm activity, to control for mu/alpha power variability due to electrode impedance and scalp thickness^89^. Trials were considered as outliers and were removed if the PSD ratio value exceeded three scaled median absolute deviations (MADs) from the median PSD ratio value (of that cluster). The average PSD ratio value of the different trials was calculated for each condition, and separately for occipital and central electrodes. The average PSD ratio for the five central electrodes represented the mu value, and the average PSD ratio for the three occipital electrodes represented the alpha value.

Finally, a log transform was applied for the average PSD ratio values as ratio data is non-normal. Taken together, a log ratio greater than 0 indicates increased mu synchronization (ERS, event-related synchronization), a log ratio less than zero indicates mu-ERD (event-related desynchronization) and a log ratio of 0 indicates zero change of mu^39,46^. Our hypotheses were formulated as expecting more ERD. This means we expected a lower number on the presented log power ratios.

## Supplementary Information

Supplementary Information.

## Data Availability

All raw and pre-processed data and scripts will be made available upon publication on the OSF project page (https://osf.io/dsfgv/). The experimental stimuli are available upon request.
