# Supplementary material for: Neural responses to emotional displays by politicians: differential mu and alpha suppression patterns in response to in-party and out-party leaders
Source: Sci Rep. 2025 Mar 11;15:8431. doi: 10.1038/s41598-025-92898-6 (PMC11897177; doi:10.1038/s41598-025-92898-6)
Supplement: Supplementary file 1 — Supplementary Information. [file 41598_2025_92898_MOESM1_ESM.pdf]

# Supplementary Material

# Neural Responses to Emotional Displays by Politicians: Differential Mu and Alpha Suppression Patterns in Response to In-Party and Out-Party Leaders

## Contents


**1 Power Analysis** **3**

1.1 Control variables . . . . . . . . . . . . . . . . . . . . . . . . . . . . . 4

**2 Descriptive Statistics** **6**

2.1 Main variables . . . . . . . . . . . . . . . . . . . . . . . . . . . . . 6

2.2 Party preferences . . . . . . . . . . . . . . . . . . . . . . . . . . . . 6

**3 Preregistered Hypothesis 1-4** **8**

3.1 Mu . . . . . . . . . . . . . . . . . . . . . . . . . . . . . . . . . . . 8

3.2 Alpha . . . . . . . . . . . . . . . . . . . . . . . . . . . . . . . . . 12

**4 Preregistered Hypothesis 5 & 6** **15**

4.1 Mu . . . . . . . . . . . . . . . . . . . . . . . . . . . . . . . . . . . 15

4.2 Alpha . . . . . . . . . . . . . . . . . . . . . . . . . . . . . . . . . 17

4.3 Bayes Factors . . . . . . . . . . . . . . . . . . . . . . . . . . . . . 19

**5 Additional Analyses** **21**

5.1 Full Models . . . . . . . . . . . . . . . . . . . . . . . . . . . . . . 21

5.2 Fixation cross baseline models . . . . . . . . . . . . . . . . . . . . . . . . . . 23

5.3 Topoplot . . . . . . . . . . . . . . . . . . . . . . . . . . . . . . . . . . . . . 25

# 1 Power Analysis

Only subject with no neurological disorders and normal or corrected-to-normal vision were allowed to participate. Subjects were recruited through the online lab website and were rewarded with 4 hours of research credits or 45 euros for the full EEG study including two other (unrelated) experiments.

To determine the sample size, an a-priori power analysis was conducted by creating a power contour plot using the online tool (see https://shiny.york.ac.uk/powercontours/) of Baker et al. (2020). In June 20201 we did a pilot test (N=9) of the current experiment. Based on this data, we calculated the mean difference between mu suppression in response to in-party and out-party emotional displays ($M_{dif} = 0.05$ mean log ratio of mu power). We furthermore calculated the within- (0.21) and between-subject standard deviation (0.11) and applied this to the online power contour tool, resulting in Figure S1. To test hypothesis 1 and 2, we will compare mu suppression of 48 trials of emotional displays of politicians against the baseline (the first 2000 ms, i.e. the still neutral image). To test hypothesis 3 and 4, we will compare mu suppression in response to the in-party and out-party condition, each consisting of 24 trials. According to Figure S1, using 24 trials per condition (per participant) with a power of 80% would require a sample size of N = 40. However, since some trials might be excluded due to disruptions during the EEG measurement, we will collect a sample size of N = 50. This will also allow us to make comparisons between emotion conditions (using 8 trials) with enough statistical power.

Finally, since our pilot study only contained a small amount of participants, we also created a power contour plot based on the data of Krivan et al. (2020), who use a similar experimental design. We applied the mean difference of mu suppression between the observation and execution task ($M_{dif} = 0.07$ mean log ratio of mu power), and the within-subject standard deviation of 0.39 and between-subject deviation of 0.11 in the power contour plot, leading to a similar required sample size of 40 to 50 participants.

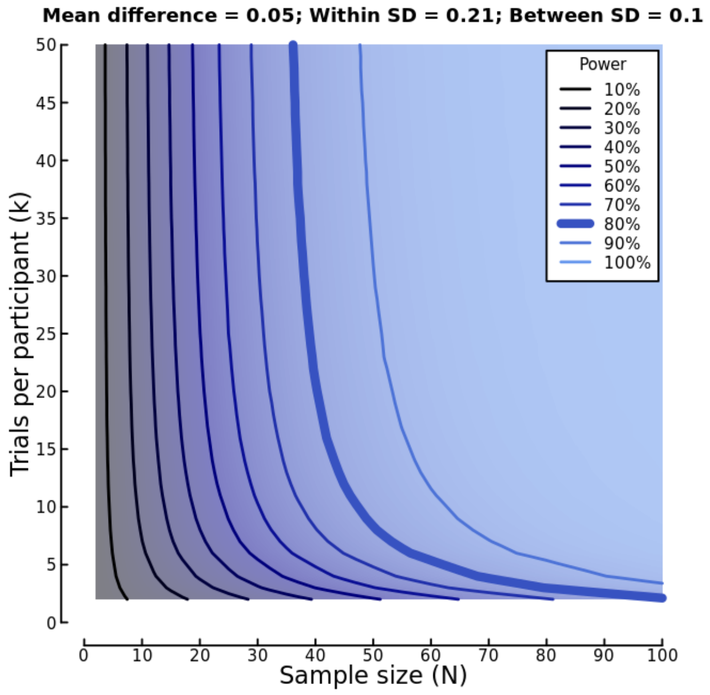


Figure S1: Power contour plot based on pilot data of the current experiment, showing lines for different amounts of power for different sample sizes (x-axis) and trials per participant (y-axis).

## 1.1 Control variables

We included several control variables in our statistical models. First of all, we control for gender (male as reference category) and party attachment with feeling thermometers (see Method section manuscript). Furthermore, we control for trait empathy and political engagement (see below). In addition, we preregistered to include a dummy variable representing any noteworthy events happening during the experiment (e.g. the participant coughing, asking a question during trial). However, this data was already filtered out in our pre-processing procedure, so the dummy variable did not add any value in our analysis and was therefore not included.

**Partisanship.** We measured party attachment with feeling thermometers, which give a good indication of the extent of participants' negative or positive feelings towards a certain party (Rosema and Mayer, 2020). After participants indicated their two in-

parties and two out-parties, participants were asked "how negative - positive do you feel about the following political party?". Four sliders were presented, each showing the name of one of the four assigned parties in random order, with 0 (very negative) to 100 (very positive), with the slider set at midpoint.

**Trait Empathy.** A short 22-item version of the Empathy Quotient (EQ Lawrence et al., 2004), EQ-Short of Wakabayashi et al. (2006) will be used. The questionnaire includes items like "I really enjoy caring for other people", with a 4-point response scale (1 = strongly disagree, 4 = strongly agree).

**Political Engagement** A one item question is included in the pre-test survey to measure participants political engagement. The question is "How often do you follow up on political news?", with a scale from 0-100 (0 = Not often at all, 100 = Very often).

# 2 Descriptive Statistics

## 2.1 Main variables

Table S1: Mean and standard deviation of mu and alpha per group (in-party, out-party, and non-politicians)

| | In-party | | Non-politician | | Out-party | |
|---|---|---|---|---|---|---|
| | mean | sd | mean | sd | mean | sd |
| mu | −0.310 | 0.109 | −0.309 | 0.119 | −0.331 | 0.145 |
| alpha | −0.317 | 0.126 | −0.320 | 0.150 | −0.346 | 0.158 |

Table S2: Mean and standard deviation of mu and alpha for the emotion conditions per group (in-party, out-party, and non-politicians)

| | | Angry | | Happy | | Neutral | |
|---|---|---|---|---|---|---|---|
| | Group | mean | sd | mean | sd | mean | sd |
| mu | In-party | −0.306 | 0.121 | −0.311 | 0.103 | −0.313 | 0.103 |
| | Non-politician | −0.304 | 0.121 | −0.313 | 0.135 | −0.310 | 0.100 |
| | Out-party | −0.340 | 0.151 | −0.339 | 0.121 | −0.316 | 0.162 |
| alpha | In-party | −0.319 | 0.120 | −0.305 | 0.127 | −0.328 | 0.130 |
| | Non-politician | −0.315 | 0.150 | −0.327 | 0.155 | −0.317 | 0.147 |
| | Out-party | −0.343 | 0.175 | −0.351 | 0.125 | −0.342 | 0.170 |

## 2.2 Party preferences

Table S3: First in-party choice (rows) and second in-party choice (columns) of participants

| | CDA | D66 (Democraten 66) | VVD |
|---|---|---|---|
| ChristenUnie | 0 | 6 | 0 |
| D66 (Democraten 66) | 6 | 0 | 24 |
| GroenLinks | 0 | 30 | 0 |
| PvdA (Partij van de Arbeid) | 0 | 6 | 0 |
| Volt | 0 | 6 | 0 |

Table S4: First out-party choice (rows) and second out-party choice (columns) of participants

| | 50PLUS | CDA | FvD | PVV | SGP | VVD |
|---|---|---|---|---|---|---|
| BIJ1 | 0 | 0 | 0 | 6 | 0 | 0 |
| ChristenUnie | 0 | 0 | 0 | 6 | 0 | 0 |
| DENK | 0 | 0 | 0 | 6 | 0 | 0 |
| Forum voor Democratie (FvD) | 6 | 6 | 0 | 24 | 12 | 6 |
| JA21 | 0 | 0 | 6 | 0 | 0 | 0 |

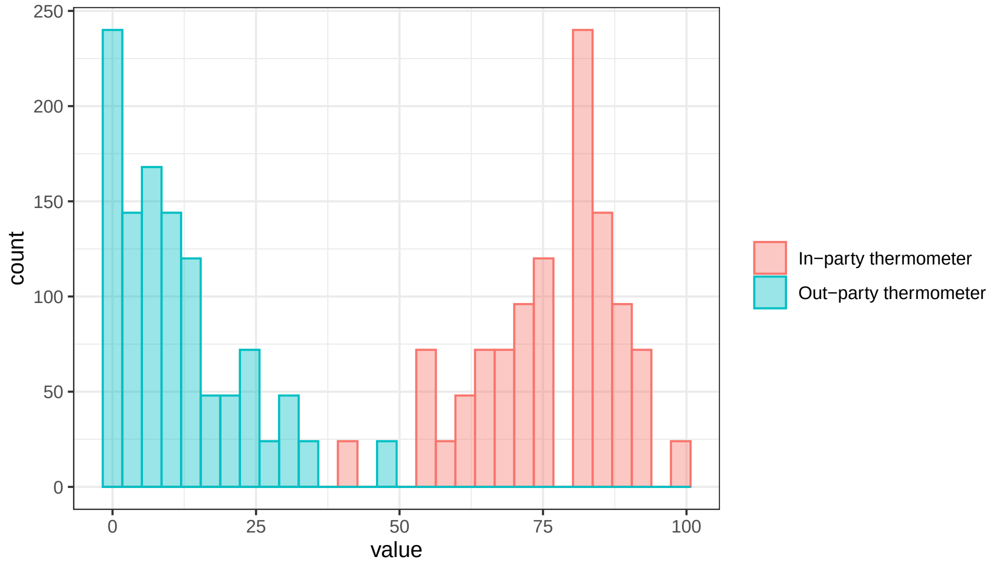


Figure S2: Distribution of in-party and out-party feeling thermometers ranging from 0 - very negative, to 100 - very positive.

# 3 Preregistered Hypothesis 1-4

## 3.1 Mu

Table S5: Preregistered OLS regression analysis of mu ERD of hypothesis 1 & 2 with in-party condition as reference category

| | All conditions | Angry | Happy | Neutral |
|---|---|---|---|---|
| (Intercept) | −0.360*** | −0.261** | −0.331** | −0.489*** |
| | (0.101) | (0.096) | (0.124) | (0.112) |
| In-party vs Non-politician | 0.001 | 0.001 | −0.001 | 0.003 |
| | (0.007) | (0.012) | (0.014) | (0.014) |
| In-party vs Out-party politician | −0.021* | −0.034* | −0.028 | −0.002 |
| | (0.009) | (0.016) | (0.018) | (0.017) |
| Age | −0.002 | −0.002*** | −0.001 | −0.002 |
| | (0.001) | (0.001) | (0.002) | (0.001) |
| Male | 0.008 | 0.006 | 0.001 | 0.016 |
| | (0.015) | (0.019) | (0.016) | (0.015) |
| Political engagement | 0.001* | 0.001 | 0.002*** | 0.001* |
| | (0.000) | (0.001) | (0.000) | (0.000) |
| Empathy | 0.014 | 0.006 | 0.002 | 0.033 |
| | (0.029) | (0.028) | (0.035) | (0.030) |
| In-party thermometer | 0.000 | 0.000 | −0.001 | 0.001 |
| | (0.001) | (0.001) | (0.001) | (0.001) |
| Out-party thermometer | 0.000 | −0.001 | 0.000 | 0.001 |
| | (0.001) | (0.001) | (0.001) | (0.001) |
| Num.Obs. | 1104 | 368 | 368 | 368 |
| R2 | 0.039 | 0.039 | 0.056 | 0.068 |
| R2 Adj. | 0.032 | 0.017 | 0.035 | 0.047 |

Standard errors are clustered at the individual level

* $p < 0.05$, ** $p < 0.01$, *** $p < 0.001$

Table S6: Preregistered OLS regression analysis of mu ERD of hypothesis 1 & 2 with non-politician condition as reference category

| | All conditions | Angry | Happy | Neutral |
|---|---|---|---|---|
| (Intercept) | −0.359*** | −0.259** | −0.353* | −0.486*** |
| | (0.099) | (0.094) | (0.137) | (0.110) |
| Non-politician vs In-party politician | −0.001 | −0.001 | 0.021 | −0.003 |
| | (0.007) | (0.012) | (0.018) | (0.014) |
| Non-politician vs Out-party politician | −0.022* | −0.035 | −0.024 | −0.006 |
| | (0.009) | (0.020) | (0.015) | (0.016) |
| Age | −0.002 | −0.002*** | −0.002 | −0.002 |
| | (0.001) | (0.001) | (0.001) | (0.001) |
| Male | 0.008 | 0.006 | 0.001 | 0.016 |
| | (0.015) | (0.019) | (0.024) | (0.015) |
| Political engagement | 0.001* | 0.001 | 0.001 | 0.001* |
| | (0.000) | (0.001) | (0.001) | (0.000) |
| Empathy | 0.014 | 0.006 | 0.011 | 0.033 |
| | (0.029) | (0.028) | (0.036) | (0.030) |
| In-party thermometer | 0.000 | 0.000 | 0.000 | 0.001 |
| | (0.001) | (0.001) | (0.001) | (0.001) |
| Out-party thermometer | 0.000 | −0.001 | 0.001 | 0.001 |
| | (0.001) | (0.001) | (0.001) | (0.001) |
| Num.Obs. | 1104 | 368 | 368 | 368 |
| R2 | 0.039 | 0.039 | 0.033 | 0.068 |
| R2 Adj. | 0.032 | 0.017 | 0.012 | 0.047 |

* $p < 0.05$, ** $p < 0.01$, *** $p < 0.001$

Standard errors are clustered at the individual level

Table S7: OLS regression analysis of mu ERD between neutral and emotion conditions per experimental group (H3 & 4)

| | In-party | Non-politician | Out-party |
|---|---|---|---|
| (Intercept) | −0.470*** | −0.302** | −0.376** |
| | (0.098) | (0.113) | (0.142) |
| Neutral vs Angry | 0.008 | 0.006 | −0.024 |
| | (0.013) | (0.011) | (0.019) |
| Neutral vs Happy | 0.002 | −0.003 | −0.024 |
| | (0.014) | (0.010) | (0.020) |
| Age | −0.001 | −0.001 | −0.003** |
| | (0.001) | (0.001) | (0.001) |
| Male | 0.005 | 0.003 | 0.018 |
| | (0.015) | (0.015) | (0.025) |
| Political engagement | 0.001* | 0.001 | 0.001* |
| | (0.000) | (0.000) | (0.001) |
| Empathy | 0.027 | 0.002 | 0.024 |
| | (0.027) | (0.031) | (0.040) |
| In-party thermometer | 0.001 | 0.000 | 0.000 |
| | (0.001) | (0.001) | (0.001) |
| Out-party thermometer | 0.001 | 0.000 | 0.000 |
| | (0.001) | (0.001) | (0.002) |
| Num.Obs. | 276 | 552 | 276 |
| R2 | 0.054 | 0.023 | 0.061 |
| R2 Adj. | 0.026 | 0.009 | 0.033 |

Standard errors are clustered at the individual level

* $p < 0.05$, ** $p < 0.01$, *** $p < 0.001$

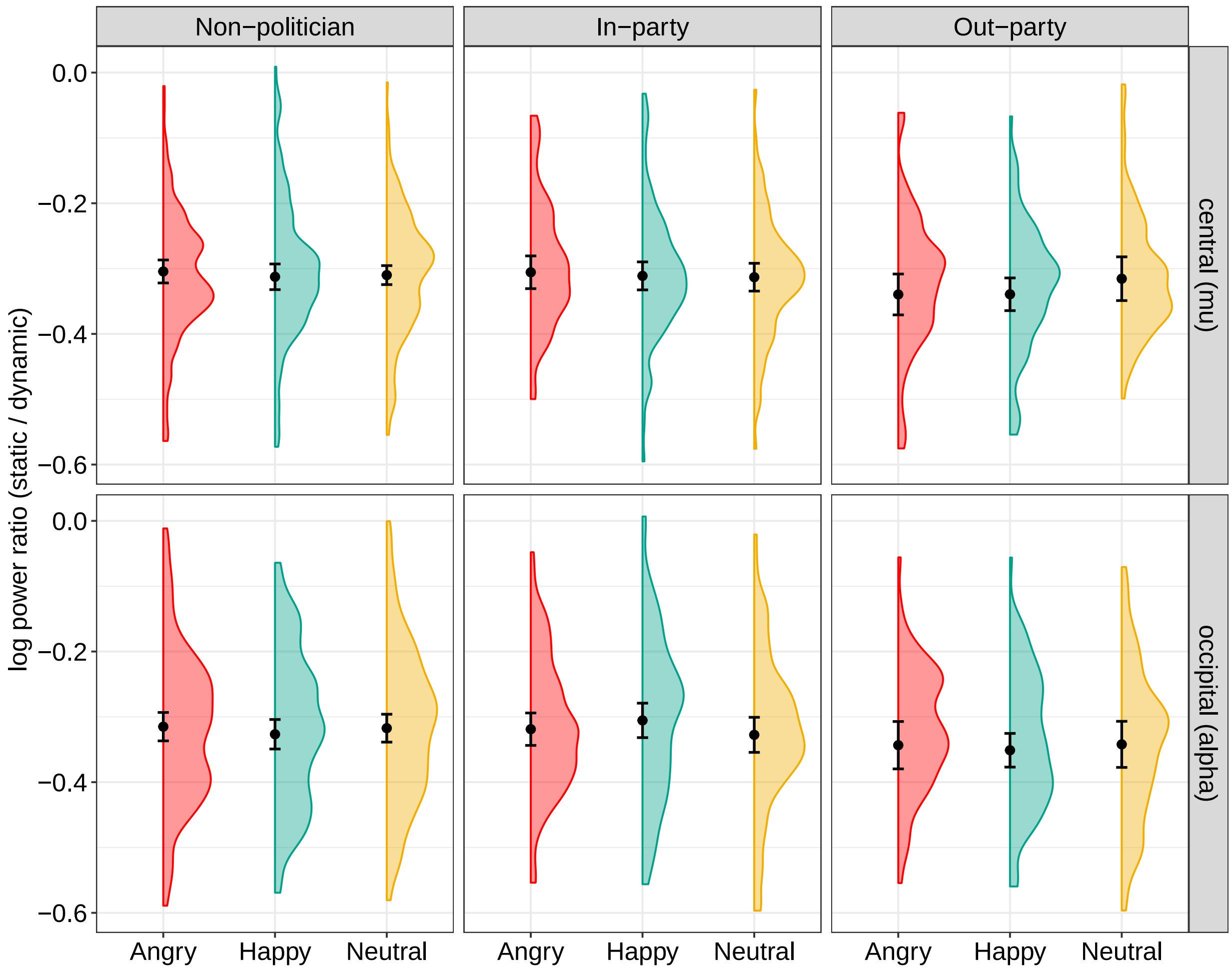


Figure S3: Log power (spectral density) ratio (static phase first 2000 ms / dynamic phase second 2000 ms) of 47 participants (Obs = 1104) over the central (mu) and occipital (alpha) electrodes for the different emotion conditions: angry (in red), happy (in green), and neutral (in yellow), for each politician condition (left: non-politician, middle: in-party, right: out-party). Black dots represents the mean estimate per group, the black whiskers the 95% confidence intervals, and the clouds the sample distributions. P-values are given for significant differences between conditions.

## 3.2 Alpha

Table S8: Preregistered OLS regression analysis of alpha ERD of hypothesis 1 & 2 with in-party condition as reference category

| | All conditions | Angry | Happy | Neutral |
|---|---|---|---|---|
| (Intercept) | −0.391*** | −0.370*** | −0.332* | −0.471*** |
| | (0.084) | (0.103) | (0.135) | (0.114) |
| In-party vs Non-politician | −0.002 | 0.004 | −0.021 | 0.010 |
| | (0.010) | (0.017) | (0.018) | (0.018) |
| In-party vs Out-party politician | −0.028* | −0.025 | −0.046* | −0.015 |
| | (0.012) | (0.016) | (0.019) | (0.022) |
| Age | −0.001 | −0.001 | −0.002 | −0.001 |
| | (0.001) | (0.001) | (0.001) | (0.002) |
| Male | −0.005 | −0.006 | 0.001 | −0.010 |
| | (0.022) | (0.025) | (0.024) | (0.027) |
| Political engagement | 0.001 | 0.000 | 0.001 | 0.001 |
| | (0.000) | (0.001) | (0.001) | (0.001) |
| Empathy | 0.017 | 0.013 | 0.011 | 0.027 |
| | (0.027) | (0.030) | (0.036) | (0.032) |
| In-party thermometer | 0.000 | 0.000 | 0.000 | 0.000 |
| | (0.001) | (0.001) | (0.001) | (0.001) |
| Out-party thermometer | 0.001 | 0.001 | 0.001 | 0.001 |
| | (0.001) | (0.001) | (0.001) | (0.001) |
| Num.Obs. | 1104 | 368 | 368 | 368 |
| R2 | 0.022 | 0.019 | 0.033 | 0.028 |
| R2 Adj. | 0.015 | −0.003 | 0.012 | 0.007 |

Standard errors are clustered at the individual level

* p < 0.05, ** p < 0.01, *** p < 0.001

Table S9: Preregistered OLS regression analysis of alpha ERD of hypothesis 1 & 2 with non-politician condition as reference category

| | All conditions | Angry | Happy | Neutral |
|---|---|---|---|---|
| (Intercept) | −0.419*** | −0.394*** | −0.377** | −0.485*** |
| | (0.085) | (0.104) | (0.139) | (0.117) |
| Non-politician vs In-party politician | 0.028* | 0.025 | 0.046* | 0.015 |
| | (0.012) | (0.016) | (0.019) | (0.022) |
| Non-politician vs Out-party politician | 0.026** | 0.028 | 0.024 | 0.025 |
| | (0.010) | (0.020) | (0.015) | (0.022) |
| Age | −0.001 | −0.001 | −0.002 | −0.001 |
| | (0.001) | (0.001) | (0.001) | (0.002) |
| Male | −0.005 | −0.006 | 0.001 | −0.010 |
| | (0.022) | (0.025) | (0.024) | (0.027) |
| Political engagement | 0.001 | 0.000 | 0.001 | 0.001 |
| | (0.000) | (0.001) | (0.001) | (0.001) |
| Empathy | 0.017 | 0.013 | 0.011 | 0.027 |
| | (0.027) | (0.030) | (0.036) | (0.032) |
| In-party thermometer | 0.000 | 0.000 | 0.000 | 0.000 |
| | (0.001) | (0.001) | (0.001) | (0.001) |
| Out-party thermometer | 0.001 | 0.001 | 0.001 | 0.001 |
| | (0.001) | (0.001) | (0.001) | (0.001) |
| Num.Obs. | 1104 | 368 | 368 | 368 |
| R2 | 0.022 | 0.019 | 0.033 | 0.028 |
| R2 Adj. | 0.015 | −0.003 | 0.012 | 0.007 |

* $p < 0.05$, ** $p < 0.01$, *** $p < 0.001$

Standard errors are clustered at the individual level

Table S10: Preregistered OLS regression analysis of alpha ERD between neutral and emotion conditions per experimental group (H3 & 4)

| | In-party | Non-politician | Out-party |
|---|---|---|---|
| (Intercept) | −0.515*** | −0.347** | −0.389** |
| | (0.116) | (0.109) | (0.137) |
| Neutral vs Angry | 0.009 | 0.002 | −0.001 |
| | (0.019) | (0.015) | (0.021) |
| Neutral vs Happy | 0.022 | −0.009 | −0.009 |
| | (0.019) | (0.012) | (0.023) |
| Age | −0.002 | −0.001 | −0.001 |
| | (0.001) | (0.001) | (0.001) |
| Male | −0.003 | −0.015 | 0.011 |
| | (0.018) | (0.026) | (0.030) |
| Political engagement | 0.001 | 0.000 | 0.001 |
| | (0.001) | (0.001) | (0.001) |
| Empathy | 0.029 | 0.020 | −0.003 |
| | (0.027) | (0.032) | (0.036) |
| In-party thermometer | 0.001 | −0.001 | 0.000 |
| | (0.001) | (0.001) | (0.001) |
| Out-party thermometer | 0.001 | 0.001 | 0.001 |
| | (0.001) | (0.001) | (0.002) |
| Num.Obs. | 276 | 552 | 276 |
| R2 | 0.057 | 0.018 | 0.015 |
| R2 Adj. | 0.029 | 0.003 | −0.014 |

Standard errors are clustered at the individual level

* $p < 0.05$, ** $p < 0.01$, *** $p < 0.001$

# 4 Preregistered Hypothesis 5 & 6

## 4.1 Mu

Table S11: Mean and Standard Deviation of mu-ERD in the static and dynamic phase (for angry, happy, and neutral conditions separate, and for all emotion conditions together) for all Group conditions and for Politicians (in- and out-party politicians)

| | | Static | | Dynamic | |
|---|---|---|---|---|---|
| | Emotion | mean | sd | mean | sd |
| All groups | Angry | 0.104 | 0.876 | -0.233 | 0.870 |
| | Happy | 0.060 | 0.830 | -0.285 | 0.775 |
| | Neutral | 0.050 | 0.771 | -0.270 | 0.803 |
| | Emotions combined | 0.072 | 0.827 | -0.263 | 0.817 |
| Politicians | Angry | 0.083 | 0.855 | -0.252 | 0.825 |
| | Happy | 0.100 | 0.910 | -0.246 | 0.885 |
| | Neutral | 0.062 | 0.799 | -0.272 | 0.818 |
| | Emotions combined | 0.082 | 0.855 | -0.256 | 0.842 |

Table S12: Results t-tests of difference in mu-ERD between the static and dynamic phase (for all emotion conditions together, and angry, happy, and neutral conditions separate)

| | Emotions combined | Angry | Happy | Neutral |
|---|---|---|---|---|
| mean difference | 0.334 | 0.337 | 0.345 | 0.320 |
| t | 65.723 | 37.968 | 36.796 | 39.482 |
| ci.lower | 0.324 | 0.319 | 0.327 | 0.304 |
| ci.upper | 0.344 | 0.354 | 0.364 | 0.336 |
| p.value | 0.000 | 0.000 | 0.000 | 0.000 |

Table S13: Results t-tests of difference in mu-ERD between the static and dynamic phase (for politician conditions with all emotion conditions combined, and angry, happy, and neutral conditions separate)

| | Politicians | Angry Politicians | Happy Politicians | Neutral Politicians |
|---:|---:|---:|---:|---:|
| mean difference | 0.338 | 0.335 | 0.346 | 0.334 |
| t | 47.830 | 27.144 | 24.588 | 33.175 |
| ci.lower | 0.324 | 0.311 | 0.318 | 0.314 |
| ci.upper | 0.352 | 0.359 | 0.374 | 0.354 |
| p.value | 0.000 | 0.000 | 0.000 | 0.000 |

## 4.2 Alpha

Table S14: Mean and Standard Deviation of alpha-ERD in the static and dynamic phase (for angry, happy, and neutral conditions separate, and for all emotion conditions together) for all Group conditions and for Politicians (in- and out-party politicians)

| | | Static | | Dynamic | |
|---|---|---|---|---|---|
| | Emotion | mean | sd | mean | sd |
| All groups | Angry | 0.041 | 0.890 | -0.312 | 0.886 |
| | Happy | -0.005 | 0.844 | -0.360 | 0.794 |
| | Neutral | -0.011 | 0.801 | -0.350 | 0.828 |
| | Emotions combined | 0.008 | 0.846 | -0.340 | 0.837 |
| Politicians | Angry | 0.032 | 0.869 | -0.324 | 0.845 |
| | Happy | 0.029 | 0.918 | -0.324 | 0.893 |
| | Neutral | -0.002 | 0.820 | -0.352 | 0.840 |
| | Emotions combined | 0.020 | 0.870 | -0.333 | 0.860 |

Table S15: Results t-tests of difference in alpha-ERD between the static and dynamic phase (for all emotion conditions together, and angry, happy, and neutral conditions separate)

| | Emotions combined | Angry | Happy | Neutral |
|---|---|---|---|---|
| mean difference | 0.349 | 0.353 | 0.354 | 0.339 |
| t | 66.275 | 37.765 | 37.149 | 40.226 |
| ci.lower | 0.338 | 0.334 | 0.336 | 0.322 |
| ci.upper | 0.359 | 0.371 | 0.373 | 0.355 |
| p.value | 0.000 | 0.000 | 0.000 | 0.000 |

Table S16: Results t-tests of difference in alpha-ERD between the static and dynamic phase (for politician conditions with all emotion conditions combined, and angry, happy, and neutral conditions separate)

| | Politicians | Angry Politicians | Happy Politicians | Neutral Politicians |
|---|---|---|---|---|
| mean difference | 0.353 | 0.357 | 0.353 | 0.350 |
| t | 48.876 | 28.165 | 24.980 | 33.166 |
| ci.lower | 0.339 | 0.332 | 0.325 | 0.329 |
| ci.upper | 0.367 | 0.382 | 0.381 | 0.370 |
| p.value | 0.000 | 0.000 | 0.000 | 0.000 |

## 4.3 Bayes Factors

Table S17: Bayes Factors (BF01) for hypotheses 1-2 and 5-6 for mu and alpha

| | Measure | All conditions | Angry | Happy | Neutral |
|---|---|---|---|---|---|
| Politicians | mu | <0.001 | <0.001 | <0.001 | <0.001 |
| | alpha | <0.001 | <0.001 | <0.001 | <0.001 |
| Non-politicians | mu | <0.001 | <0.001 | <0.001 | <0.001 |
| | alpha | <0.001 | <0.001 | <0.001 | <0.001 |
| In-party vs Out-party politician | mu | 0.977 | 0.760 | 1.908 | 6.196 |
| | alpha | 0.522 | 2.247 | 0.530 | 5.116 |
| In-party vs Non-politician | mu | 10.467 | 6.224 | 6.224 | 6.093 |
| | alpha | 10.269 | 6.107 | 3.187 | 5.336 |
| Out-party vs Non-politician | mu | 1.067 | 1.540 | 2.011 | 5.917 |
| | alpha | 0.740 | 2.444 | 1.867 | 3.433 |

Table S18: Bayes Factors (BF01) for hypotheses 3-4 for mu and alpha

| | Measure | All group conditions | In-party politicians | Out-party politicians | Non-politicians |
|---|---|---|---|---|---|
| Angry vs. Happy | mu | 9.5000 | 5.805 | 6.252 | 5.317 |
| | alpha | 10.376 | 4.828 | 5.838 | 4.821 |
| Angry vs Neutral | mu | 9.656 | 5.329 | 3.051 | 5.542 |
| | alpha | 10.095 | 5.696 | 6.241 | 6.183 |
| Happy vs Neutral | mu | 6.938 | 6.195 | 3.191 | 6.052 |
| | alpha | 10.483 | 3.233 | 5.810 | 4.743 |

Table S19: Bayes Factors *evidence for the alternative hypothesis* (BF10) of hypotheses 1-2 and 5-6 for mu and alpha. Note here that the direction of the comparisons based on the theoretical expectation, the performed comparisons are the other way around since mu ERD means lower values.

| | Measure | All conditions | Angry | Happy | Neutral |
|---|---|---|---|---|---|
| Politicians: | mu | 1.247x10+130 | 5.329x10+58 | 1.951x10+31 | 2.549x10+48 |
| static <dynamic | alpha | 1.258x10+134 | 1.033x10+60 | 1.447x10+32 | 6.225x10+48 |
| Non-politicians: | mu | 4.659x10+125 | 1.694x10+55 | 2.52x10+42 | 1.004x10+31 |
| static <dynamic | alpha | 4.223x10+123 | 1.026x10+50 | 5.011x10+41 | 2.897x10+32 |
| In-party <Out-party | mu | 2.016 | 2.576 | 0.983 | 0.179 |
| politician | alpha | 3.801 | 0.821 | 3.721 | 0.288 |
| Non-politician <Out- | mu | 1.845 | 1.237 | 0.928 | 0.288 |
| party politician | alpha | 2.675 | 0.747 | 1.006 | 0.213 |

# 5 Additional Analyses

## 5.1 Full Models

Table S20: OLS Regression Analysis of Hypothesis 1 and 2 (for all emotion conditions) of mu ERD with alpha as control (model 1), of alpha ERD with mu as control (model 2), and of mu and alpha combined with electrode location variable included

| | Model 1 (mu) | Model 2 (alpha) | Model 3 (combined) |
|---|---|---|---|
| (Intercept) | -0.203* | -0.185** | -0.379*** |
| | (0.085) | (0.070) | (0.084) |
| In-party vs Non-politician | 0.002 | -0.003 | -0.002 |
| | (0.006) | (0.009) | (0.010) |
| In-party vs Out-party politician | -0.010 | -0.016 | -0.028* |
| | (0.008) | (0.011) | (0.012) |
| Age | -0.001 | -0.000 | -0.001* |
| | (0.001) | (0.001) | (0.001) |
| Male | 0.010 | -0.010 | 0.001 |
| | (0.010) | (0.016) | (0.017) |
| Political engagement | 0.001** | -0.000 | 0.001 |
| | (0.000) | (0.000) | (0.000) |
| Empathy | 0.007 | 0.009 | 0.015 |
| | (0.022) | (0.019) | (0.026) |
| In-party thermometer | -0.000 | 0.000 | 0.000 |
| | (0.000) | (0.001) | (0.001) |
| Out-party thermometer | -0.000 | 0.001* | 0.001 |
| | (0.001) | (0.000) | (0.001) |
| Alpha | 0.402*** | | |
| | (0.079) | | |
| Mu | | 0.572*** | |
| | | (0.075) | |
| Location (alpha vs mu) | | | 0.007 |
| | | | (0.007) |
| Location*In-party vs Non-politician | | | 0.003 |
| | | | (0.010) |
| Location*In-party vs Out-party | | | 0.007 |
| | | | (0.012) |
| Num.Obs. 1104 | 1104 | 2208 | |
| R2 0.260 | 0.247 | 0.028 | |
| R2 Adj. 0.254 | 0.241 | 0.023 | |

Standard errors are clustered at the individual level

* p < 0.05, ** p < 0.01, *** p < 0.001

Table S21: OLS Regression Analysis of Hypothesis 1 and 2 without preregistered controls (model 1: DV = mu, model 3: DV = alpha) and with full group and emotion condition interaction (model 2: DV = mu, model 4: DV = alpha)

| | Model 1 | Model 2 | Model 3 | Model 4 |
|---|---|---|---|---|
| (Intercept) | -0.266*** | -0.269*** | -0.287*** | -0.297*** |
| | (0.030) | (0.030) | (0.018) | (0.021) |
| In-party vs Non-politician | 0.001 | 0.003 | -0.002 | 0.010 |
| | (0.007) | (0.014) | (0.010) | (0.018) |
| In-party vs Out-party politician | -0.021* | -0.002 | -0.028* | -0.015 |
| | (0.009) | (0.017) | (0.012) | (0.022) |
| Age | -0.002 | -0.002 | -0.001 | -0.001 |
| | (0.001) | (0.001) | (0.001) | (0.001) |
| Male | 0.008 | 0.008 | -0.006 | -0.006 |
| | (0.015) | (0.015) | (0.020) | (0.020) |
| N vs Angry | | 0.008 | | 0.009 |
| | | (0.013) | | (0.019) |
| N vs Happy | | 0.002 | | 0.022 |
| | | (0.014) | | (0.019) |
| IP vs Non-politician*N vs Angry | | -0.002 | | -0.006 |
| | | (0.018) | | (0.026) |
| IP vs Out-party*N vs Angry | | -0.032 | | -0.010 |
| | | (0.021) | | (0.024) |
| IP vs Non-politician*N vs Happy | | -0.005 | | -0.031 |
| | | (0.019) | | (0.023) |
| IP vs Out-party*N vs Happy | | -0.026 | | -0.031 |
| | | (0.027) | | (0.031) |
| Num.Obs. | 1104 | 1104 | 1104 | 1104 |
| R2 | 0.019 | 0.021 | 0.010 | 0.012 |
| R2 Adj. | 0.015 | 0.012 | 0.007 | 0.003 |

Standard errors are clustered at the individual level

* p < 0.05, ** p < 0.01, *** p < 0.001

IP = In-party politician, N = Neutral

## 5.2 Fixation cross baseline models

Table S22: Mean and Standard Deviation of mu-ERD ratio with fixation cross as baseline by Emotion and Group of the dynamic phase

| Emotion | Group | mean | sd |
|---|---|---|---|
| Angry | In-party | 0.390 | 0.559 |
| | Non-politician | 0.379 | 0.532 |
| | Out-party | 0.375 | 0.530 |
| Happy | In-party | 0.469 | 0.842 |
| | Non-politician | 0.381 | 0.543 |
| | Out-party | 0.394 | 0.623 |
| Neutral | In-party | 0.378 | 0.541 |
| | Non-politician | 0.381 | 0.551 |
| | Out-party | 0.389 | 0.523 |

Table S23: OLS Regression Analysis per Emotion condition with in-party condition as reference category using mu-ERD corrected with fixation cross as baseline

| | All conditions | Angry | Happy | Neutral |
|---|---|---|---|---|
| (Intercept) | 6.665 | 6.478 | 6.252 | 7.545 |
| | (4.066) | (4.453) | (4.605) | (4.153) |
| In-party vs Non-politician | 0.117 | 0.174 | 0.268 | -0.111 |
| | (0.139) | (0.271) | (0.211) | (0.187) |
| In-party vs Out-party politician | 0.014 | 0.171 | 0.065 | -0.262 |
| | (0.207) | (0.305) | (0.247) | (0.272) |
| Age | 0.151 | 0.139 | 0.168 | 0.147 |
| | (0.095) | (0.109) | (0.095) | (0.094) |
| Male | 0.191 | -0.090 | 0.280 | 0.397 |
| | (0.632) | (0.671) | (0.645) | (0.676) |
| Political engagement | -0.041* | -0.042* | -0.035* | -0.046* |
| | (0.017) | (0.018) | (0.016) | (0.019) |
| Empathy | -2.569* | -2.501 | -2.605* | -2.656** |
| | (1.099) | (1.305) | (1.129) | (1.007) |
| In-party thermometer | 0.002 | 0.006 | -0.001 | -0.001 |
| | (0.020) | (0.021) | (0.019) | (0.024) |
| Out-party thermometer | 0.041 | 0.045 | 0.040 | 0.039 |
| | (0.031) | (0.035) | (0.031) | (0.029) |
| Num. Obs. | 1291 | 446 | 404 | 441 |
| R2 | 0.316 | 0.272 | 0.349 | 0.369 |

## 5.3 Topoplot

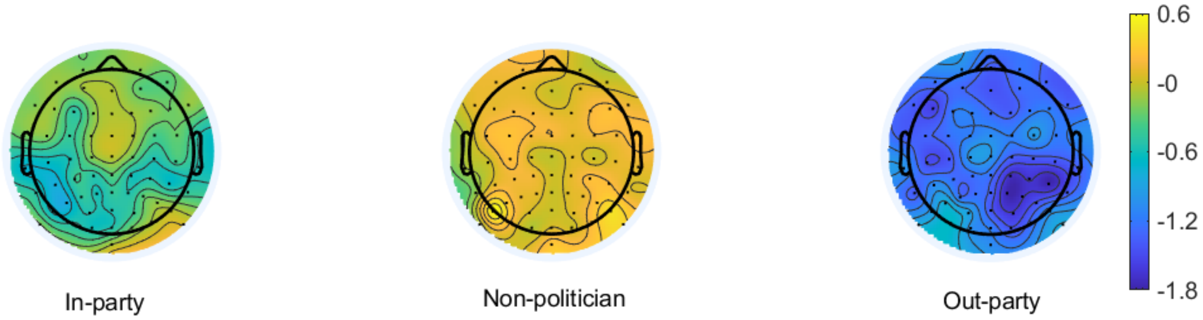


Figure S4: ERSP topoplot of mu rhythm activity (8-13 Hz) for the In-party, Non-politician and Out-party condition during the dynamic phase (2000 - 4000 ms)

# References

Baker, D. H., Vilidaite, G., Lygo, F. A., Smith, A. K., Flack, T. R., Gouws, A. D., and Andrews, T. J. (2020). Power Contours: Optimising Sample Size and Precision in Experimental Psychology and Human Neuroscience. *Psychological Methods*, 26(3):295–314.

Krivan, S. J., Caltabiano, N., Cottrell, D., and Thomas, N. A. (2020). I'll cry instead: Mu suppression responses to tearful facial expressions. *Neuropsychologia*, 143.

Lawrence, E. J., Shaw, P., Baker, D., Baron-Cohen, S., and David, A. S. (2004). Measuring empathy: reliability and validity of the Empathy Quotient. *Psychological Medicine*, 34(5):911–920.

Rosema, M. and Mayer, S. J. (2020). Measuring party attachments with survey questionnaires. In Oscarsson, H. and Holmberg, S., editors, *Research Handbook on Political Partisanship*. Edward Elgar.

Wakabayashi, A., Baron-Cohen, S., Wheelwright, S., Goldenfeld, N., Delaney, J., Fine, D., Smith, R., and Weil, L. (2006). Development of short forms of the Empathy Quotient (EQ-Short) and the Systemizing Quotient (SQ-Short). *Personality and Individual Differences*, 41(5):929–940.
